# Late pulmonary adverse effects in childhood and adolescent acute lymphoblastic leukaemia survivors: a cross-sectional ALL-STAR Lungs study

**DOI:** 10.1183/23120541.00350-2025

**Published:** 2025-12-01

**Authors:** Sonja Izquierdo Riis Meyer, Mette Tiedemann Skipper, Birgitte Klug Albertsen, Ruta Tuckuviene, Peder Skov Wehner, Thomas Leth Frandsen, Kjeld Schmiegelow, Liv Andrés-Jensen, Kim Gjerum Nielsen, Sune Leisgaard Mørck Rubak

**Affiliations:** 1 Department of Paediatrics and Adolescent Medicine, Danish Centre of Paediatric Pulmonology and Allergology, Aarhus University Hospital, Aarhus, Denmark; 2 Department of Clinical Medicine, Aarhus University, Aarhus, Denmark; 3 Department of Paediatrics and Adolescent Medicine, Centre for Paediatric and Adolescent Cancer, Aarhus University Hospital, Aarhus, Denmark; 4 Department of Paediatrics, Aalborg University Hospital, Aalborg, Denmark; 5 Department of Paediatric Haematology and Oncology, Odense University Hospital, Odense, Denmark; 6 Mary Elizabeth's Hospital – Rigshospitalet for Children, Teens and Expecting Families, Juliane Marie Centre, Copenhagen University Hospital, Rigshospitalet, Copenhagen, Denmark; 7 Department of Paediatrics and Adolescent Medicine, Juliane Marie Centre, Copenhagen University Hospital, Rigshospitalet, Copenhagen, Denmark; 8 Institute of Clinical Medicine, University of Copenhagen, Faculty of Health and Medical Sciences, Copenhagen, Denmark; 9 Department of Paediatrics and Adolescent Medicine, Danish Paediatric Pulmonary Service, Copenhagen University Hospital, Copenhagen, Denmark; 10 These authors contributed equally

## Abstract

**Background:**

Childhood acute lymphoblastic leukaemia (ALL) survival rates have improved, reaching 95%. However, 25–50% of survivors experience significant treatment-related toxicities. This study investigated late pulmonary adverse effects, including pulmonary symptoms and pulmonary function, in childhood ALL survivors compared to controls.

**Methods:**

This Danish national cross-sectional study (February 2019 to May 2024) recruited ALL survivors (n=295 eligible) and matched controls 1:1 for pulmonary symptom questionnaire and pulmonary function tests (PFTs): nitrogen multiple breath washout, spirometry, impulse oscillometry, bronchodilator response, diffusing capacity of the lung for carbon monoxide (*D*_LCO_) and nitric oxide (*D*_LNO_).

**Results:**

Among 192 survivors and 213 controls performing PFTs, 185 survivors and 209 controls had valid PFTs included in analyses. Prevalences of resting and exertional dyspnoea in survivors (2% and 14%, respectively) *versus* controls (0% and 3%, respectively) were highest among survivors treated with high-risk chemotherapy (HR) (6% and 18%, respectively) and high-risk chemotherapy followed by stem cell transplantation (HR-SCT) (5% and 26%, respectively). Compared to controls, HR and HR-SCT survivors had impaired mean z-scores for forced expiratory volume in 1 s (−1.64 and −0.37, respectively, *versus* 0.28), forced vital capacity (−1.66 and −0.67, respectively, *versus* 0.20), *D*_LNO_ (−2.39 and −2.55, respectively, *versus* 0.97) and HR-SCT survivors: lung clearance index (3.31 *versus* 0.52) and *D*_LCO_ (−1.23 *versus* 0.12).

**Conclusions:**

We found a higher reported prevalence of exertional dyspnoea and impaired pulmonary function in HR and HR-SCT survivors compared to controls. Reassuringly, non-HR/HR-SCT survivors demonstrated pulmonary function comparable to peers. The findings highlight the need for timely detection and tailored monitoring of pulmonary function deficit to improve pulmonary health in HR and HR-SCT survivors.

## Shareable abstract

**The pulmonary health of most childhood ALL survivors resembles their peers. However, those receiving high-risk therapies face a higher risk of late pulmonary adverse effects. Tailored, early pulmonary function monitoring may ensure timely detection and care.** https://bit.ly/3SaaltB

## Introduction

Acute lymphoblastic leukaemia (ALL) is the most frequent childhood malignancy, with an annual incidence rate of 4 per 100 000 children [1]. Survival rates have risen from 70% to 90% in developed countries during the past three decades [2] due to more intensive chemotherapy treatment regimens, refined risk stratification and enhanced supportive care. An overall survival rate >92% was achieved in children aged 1–17.9 years at diagnosis in the NOPHO ALL2008 protocol (the Nordic Society of Paediatric Haematology and Oncology, ALL treatment protocol recruiting from 2008 until 2018) [3]. However, the high survival rate comes at the cost of treatment-related toxicities, affecting 25–50% of survivors [4, 5].

Long-term health outcomes associated with treatment-related toxicities, such as late pulmonary adverse effects, remain a significant concern and contribute significantly to the cumulative burden in childhood cancer survivors [6]. Pulmonary toxicities can arise from various factors, including chemotherapy agents, complications from haematopoietic stem cell transplantation [7–9] and respiratory tract infections during treatment [3, 10, 11]. The latter affects ∼40% of children and can be particularly severe due to their compromised immune function [10, 11]. Treatment-related late pulmonary adverse effects that persist or emerge after treatment cessation include pulmonary function deficit and pulmonary symptoms.

Increased lung-associated standardised mortality ratios (SMRs) and absolute excess risks (AERs) per 10 000 person-years have been reported among 1) childhood and 2) adolescent and young adult leukaemia survivors in recent studies, although the rates were relatively low: 1) SMR=4.2–8.5 and AER=1.4; 2) SMR=4.8–8.1 and AER=5.0, respectively [12, 13]. However, our understanding of pulmonary symptoms and pulmonary function effects in more recent survivor generations remains limited. Previous studies have evaluated late pulmonary adverse effects in survivors treated according to older treatment regimens, including cranial irradiation (now obsolete) [14, 15], in pooled in childhood cancer survivor cohorts, only after stem cell transplantation (SCT) or in smaller cohorts [7–9, 16]. Furthermore, these previous studies used chart pulmonary function test (PFT) data or pre-existing normal range as the sole reference standard and did not include nonrelated controls. Current standard follow-up recommendations do not include routine standard PFTs for survivors without symptoms or exposure to pulmotoxic therapies or SCT [17], despite established evidence of the high yield of PFTs for screening of late pulmonary adverse effects and potential pulmonary function deficit, including restrictive, obstructive and/or diffusion defect [18].

We aimed to assess 1) pulmonary symptoms and 2) pulmonary function using multiple PFT modalities in children and adolescent ALL survivors treated according to a recent contemporary protocol and comparing them to matched healthy controls. We hypothesised that ALL survivors overall report a higher prevalence of pulmonary symptoms and exhibit impaired pulmonary function compared to age- and gender-matched controls.

## Methods

### Study design

This national cross-sectional population-based study was part of the Danish Acute Lymphoblastic Leukaemia Survivor Toxicity and Rehabilitation (ALL-STAR) study investigating subjective and objective treatment-related morbidity following ALL cure in children and young adults [19]. The ALL-STAR study included patient-reported and objective clinical outcomes following treatment according to the NOPHO ALL2008 protocol at the four Danish paediatric oncology treatment centres. Study recruitment and clinical examinations across all organ systems were performed at Copenhagen University Hospital (Copenhagen, Denmark) and Aarhus University Hospital (Aarhus, Denmark) between February 2019 and May 2024. The reporting of this study followed the Strengthening the Reporting of Observational Studies in Epidemiology guidelines [20, 21].

### Study subjects

The study population consisted of ALL survivors and controls aged 5–17.9 years at the time of examination. Eligible ALL survivors included those with Philadelphia chromosome-negative B-cell precursor or T-cell ALL, including relapse-treated survivors, who had completed treatment ≥1 year before their inclusion in the ALL-STAR study (n=295), corresponding to the NOPHO ALL2008 cohort in Denmark [19]. Community controls included cancer- and chemotherapy-naive, age- and gender-matched individuals (1:1), excluding first-degree relatives to participating survivors. They were recruited through participating survivors and social media. Study participants were survivors and controls who performed at least one PFT on the ALL-STAR examination day [19]. Participants with at least one valid PFT (defined later), and only valid PFT measures, were included in the analyses. Written informed consent was obtained from all participants’ parents or other legal guardians.

### Participant characteristics

ALL characteristics were retrieved from the NOPHO ALL2008 registry (protocol overview and description in supplementary figure S1). Characteristics included age at diagnosis and final risk group status, *i.e.* standard-risk (SR), intermediate-risk (IR), high-risk (HR) chemotherapy and high-risk chemotherapy followed by stem cell transplantation (HR-SCT). Age at examination, smoking status (both proxy-reported for participants aged <18 years, and referring to parent/caregiver report and self-reported for participants aged 15–17.9 years) and anthropometric measures (height, body mass index (BMI)) were collected through the ALL-STAR study [19]. Data on participants’ medication use and pre-existing lung diseases (*e.g.* asthma) were not assessed in this study.

### Pulmonary outcomes

#### Pulmonary symptoms

Pulmonary symptoms were assessed through electronic study-specific questionnaires designed for both proxy-reported formats (for participants aged <18 years, referring to parent/caregiver report) and self-reported formats (for participants aged 15–17.9 years). This age stratification was applied solely to accommodate the available questionnaire response formats (proxy- and self-report) in the ALL-STAR study [19]. The rationale for incorporating adolescent perspectives into the study design was informed by a prior qualitative study on survivorship challenges in adolescent and young adult ALL survivors [22], which helped shape the ALL-STAR examination programme. The questions included enquiries about resting dyspnoea, exertional dyspnoea, need of oxygen supply and everyday life impact degree of symptoms (1=not at all, 2=very little, 3=moderate, 4=severe, 5=extreme) (supplementary table S1a).

#### Pulmonary function tests

A blood sample for haemoglobin concentration (for correction of diffusing capacity) and multiple PFTs were collected on the ALL-STAR examination day (supplementary table S1a). PFTs were performed, real-time quality assessed and validated by a trained operator according to European Respiratory Society/American Thoracic Society recommendations and criteria. The PFTs included 1) nitrogen multiple breath washout test (N_2_MBW) to assess lung clearance index (LCI) [23–26]; 2) spirometry including bronchodilator response (BDR) to assess flows and reversibility [27]; 3) impulse oscillometry (IOS) including BDR to assess airway resistance, reactance and reversibility [28]; 4) total diffusing capacity of the lung for carbon monoxide (*D*_LCO_) [29]; and 4) total diffusing capacity of the lung for nitric oxide (*D*_LNO_) [30] (supplementary table S1a). All PFTs were independently validated *post hoc* by another research team member. Standard reference population and equations used for z-score calculations are detailed in supplementary table S1. Data collection at both sites was conducted by a research team comprising one or two trained research nurses, research assistants and a physician to ensure consistency and reliability. Data collection was managed using Research Electronic Data Capture (REDCap) tools hosted at the Capital Region of Denmark [31, 32].

### Statistical analyses

All statistical tests were two-sided with a significance level of 0.05 and conducted with a noncausal, descriptive approach with unadjusted estimates. Demographics were compared between survivors and controls. Outcomes were evaluated and compared across 1) ALL survivors and controls; 2) ALL survivors stratified by final risk groups (SR, IR, HR, HR-SCT) and controls; and 3) ALL survivors according to resting dyspnoea status (proxy- and/or self-reported) and exertional dyspnoea status (proxy- and/or self-reported). Additionally, demographics and treatment characteristics were compared for survivors according to study participation level and PFT validity and for ALL patients deceased or lost to follow-up. Fisher's exact test was applied to dichotomous and categorical variables. Wilcoxon rank-sum/Mann–Whitney U-test and Kruskal–Wallis test were applied to non-normally distributed continuous variables. Crude t-tests (for two-group comparisons) and one-way ANOVA (for multiple-group comparisons) were used to compare normally distributed continuous variables between groups. Age-, gender- and height-adjusted PFT z-score variables were used. Continuous variables were presented as mean±sd or median (interquartile range (IQR)) based on their distribution, while dichotomous variables were presented as frequencies and percentages. Results from comparisons were reported as mean difference (95% CI). Statistical analyses were performed using Stata/SE (version 18.0; StataCorp, College Station, TX, USA).

### Ethics

The ALL-STAR study was conducted according to the principles of the Declaration of Helsinki and approved by the regional ethics committee for the Capital Region of Denmark (H-18035090/H-20006359) and the Danish Data Protection Agency (VD-2018–519).

## Results

### Participants and demographics

**FIGURE 1:**
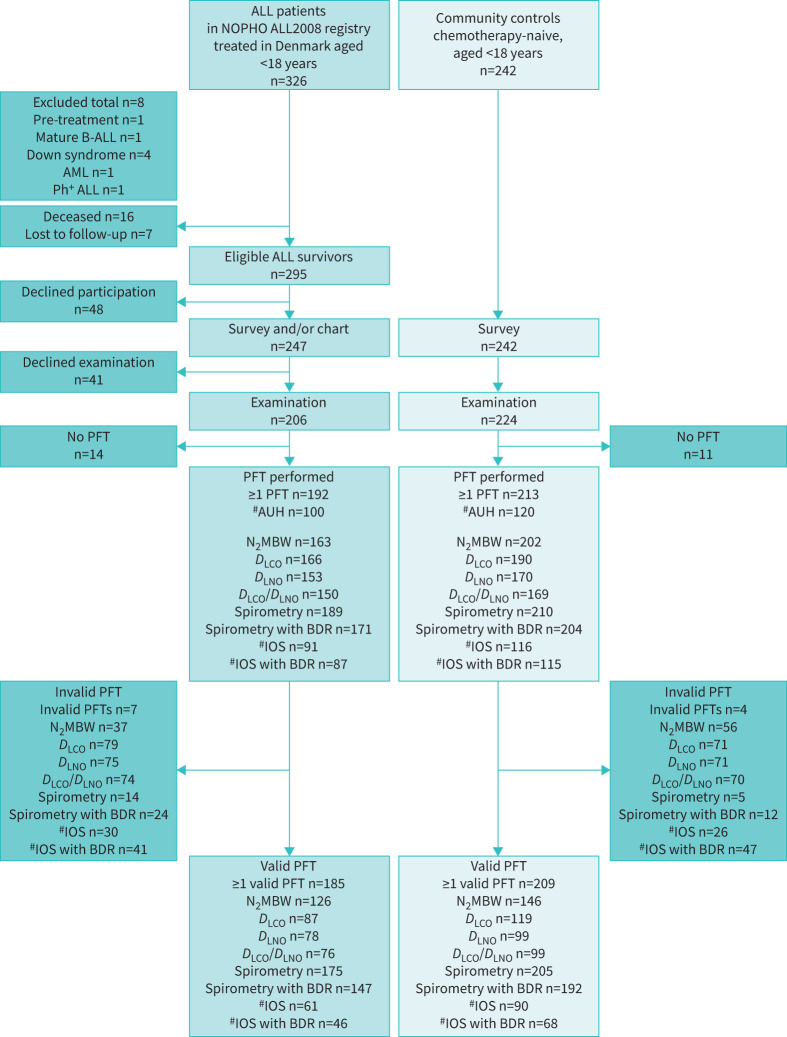
Flow diagram of Acute Lymphoblastic Leukaemia Survivor Toxicity and Rehabilitation (ALL-STAR) Lungs recruitment and participation. NOPHO ALL2008: Nordic Society of Paediatric Haematology and Oncology, acute lymphoblastic leukaemia (ALL) treatment protocol recruiting from 2008 until 2018; B-ALL: B-cell ALL; AML: acute myeloid leukaemia; Ph^+^: Philadelphia chromosome positive; PFT: pulmonary function test; AUH: Aarhus University Hospital; N_2_MBW: nitrogen multiple-breath washout test; *D*_LCO_: diffusing capacity of the lung for carbon monoxide; *D*_LNO_: diffusing capacity of the lung for nitric oxide; BDR: bronchodilator response: IOS: impulse oscillometry. ^#^: IOS was performed only at Aarhus University Hospital, Denmark. Created in BioRender. Meyer, S. (2024) https://BioRender.com/r05×459.

Among the 326 patients aged <18 years registered in the NOPHO ALL2008 registry in Denmark, 295 ALL survivors were eligible for the present study. 405 participants with at least one PFT performed were included: 192 ALL survivors and 213 controls (figure 1). Survivors with at least one PFT were shorter in mean height and height z-score (p=0.011 and p<0.001) and had a higher mean BMI z-score than controls (p=0.012). The groups were comparable regarding gender distribution (p=0.317), current smoking status (proxy-report: p=0.106; self-report: p=0.302), age (p=0.591) and BMI (p=0.110) (table 1).

**TABLE 1:** Characteristics of participants with one or more pulmonary function test (PFT) completed

	Controls	ALL survivors	p*-*value
**≥1 PFT, age <18 years**	213	192	
**≥1 PFT, age 15–17.9 years**	34	27	
**Male sex**	125 (58.7)	103 (53.6)	0.317^§^
**Smoking, proxy-report** ^#^	0 (0.0)	3 (1.6)	0.106^§^
**Smoking, self-report** ^¶^ **^,^** ^+^	1 (2.9)	3 (12.0)	0.302^§^
**Age at examination years**	11.9±2.9	11.8±3.0	0.591^ƒ^
**Height cm**	155.2±17.2	150.8±17.4	0.011^ƒ^
**Height z-score**	0.37±1.04	−0.13±1.15	<0.001^ƒ^
**BMI z-score**	0.15±1.02	0.44±1.28	0.012^ƒ^
**BMI kg·m^−2^**	17.6 (16.1–19.8)	18.0 (16.4–20.5)	0.110^##^

Data are presented as n, n (%), mean±sd or median (interquartile range), unless otherwise stated. ALL: acute lymphoblastic leukaemia; BMI: body mass index. ^#^: responses provided by a parent/caregiver for participants aged <18 years; ^¶^: responses provided by participants aged 15–17.9 years; ^+^: demographic data were missing for participants regarding self-reported smoking status (aged 15–17.9 years) for two ALL survivors with at least one PFT done; percentage of participants aged ≥15 years with at least one PFT and self-reported smoking questionnaire: controls n=34, ALL survivors n=25; ^§^: Fisher's exact test of equality; ^ƒ^: unpaired t-test of equality; ^##^: Wilcoxon rank-sum test/Mann–Whitney U-test.

### Pulmonary symptoms

Among participants with at least one valid PFT, 185 ALL survivor and 208 control proxy-report questionnaires (age <18 years) and 26 ALL survivor and 34 control self-report questionnaires (age 15–17.9 years) were completed (tables 2 and 3).

**TABLE 2:** Comparison of pulmonary symptoms and function between acute lymphoblastic leukaemia (ALL) survivors and controls at least one valid pulmonary function test (PFT)

	Participants	Controls	Participants	ALL survivors	p-value
**≥1 valid PFT, age <18 years**	209		185		
**≥1 valid PFT, age 15–17.9 years**	34		27		
**Proxy-reported symptoms, age <18 years** ^#^	208		185		
Resting dyspnoea		0 (0.0)		3 (1.6)	0.10^##^
Exertional dyspnoea		7 (3.4)		26 (14.1)	<0.001^##^
**Self-reported symptoms, age 15–17.9 years** ^¶^	34		26		
Resting dyspnoea^+^		0 (0)		1 (4)	0.43^##^
Exertional dyspnoea^+^		0 (0)		2 (8)	0.18^##^
**Proxy-reported symptoms, age <18 years** ^#^					
Exertional dyspnoea, degree 1–5^§^	7	1.9±0.7	26	3.0±0.7	0.001^¶¶^
**N_2_MBW, valid**					
LCI 2.5%^ƒ^	146	6.56±0.62	126	6.71±0.86	0.091^¶¶^
LCI 2.5% z-score^ƒ^	146	0.52±1.75	126	0.91±2.65	0.15^¶¶^
**Spirometry, valid**					
FEV_1_ z-score	205	0.28±1.06	175	−0.07±1.19	0.003^¶¶^
FVC z-score	205	0.20±0.98	175	−0.19±1.15	<0.001^¶¶^
FEV_1_/FVC z-score	205	0.12±1.02	175	0.21±1.04	0.41^¶¶^
**Spirometry with BDR**					
FEV_1_% BDR	192	4.26±4.30	147	4.32±4.37	0.88^¶¶^
MMEF_75/25_ % BDR	192	13.60±12.41	147	14.97±15.79	0.37^¶¶^
**IOS, valid**					
*R*_5Hz_ z-score	90	−0.00±0.43	61	0.06±0.50	0.40^¶¶^
*X*_5Hz_ z-score	90	0.24±0.81	61	0.15±0.97	0.55^¶¶^
**IOS with BDR, valid**					
*R*_5Hz_ % BDR	68	−15.22±13.93	46	−17.37±10.79	0.38^¶¶^
*X*_5Hz_ % BDR	68	−12.78±24.67	46	−19.83±14.58	0.084^¶¶^
***D*_LCO_, valid**					
*D*_LCO_ z-score	119	0.12±0.90	87	−0.30±0.85	<0.00^¶¶^
***D*_LNO_, valid**					
*D*_LNO_ z-score	99	0.97±2.79	78	−1.57±2.90	<0.00^¶¶^
***D*_LNO_/*D*_LCO_, valid**					
*D*_LNO_/*D*_LCO_ ratio z-score	99	3.92±4.72	76	0.74±3.81	<0.00^¶¶^
**IOS and IOS with BDR, valid**					
*D*_5–20%_	90	9.20 (2.86–17.76)	61	15.67 (7.57–24.69)	0.009^++^
*D*_5–20%_ % BDR	67	−17.00 (−68.00–34.00)	45	−20.00 (−43.00–19.00)	0.69^++^

Data are presented as n, n (%), mean±sd or median (interquartile range), unless otherwise stated. N_2_MBW: nitrogen multiple-breath washout test; LCI: lung clearance index; FEV_1_: forced expiratory volume in 1 s; FVC: forced vital capacity; BDR: bronchodilator response; MMEF_75/25_: forced expiratory flow between 25% and 75% of FVC; IOS: impulse oscillometry; *R*_5Hz_: airway resistance at a frequency of 5 Hz; *X*_5Hz_: airway reactance at a frequency of 5 Hz; *D*_LCO_: diffusing capacity of the lung for carbon monoxide; *D*_LNO_: diffusing capacity of the lung for nitric oxide; *D*_5–20%_: the difference between *R*_5Hz_ and *R*_20Hz_. ^#^: responses provided by a parent/caregiver for participants aged <18 years; ^¶^: responses provided by participants aged 15–17.9 years; ^+^: percentage of participants aged >15 years with at least one valid lung test and questionnaire; ^§^: 1=not at all, 2=very little, 3=moderate, 4=severe, 5=extreme; ^ƒ^: pooled N_2_MBW data from SentrySuite version 3.1.6, 3.2.1 and 3.3.1/3.3.2; ^##^: Fisher's exact test of equality; ^¶¶^: unpaired t-test of equality; ^++^: Wilcoxon rank-sum test/Mann–Whitney U-test.

**TABLE 3:** Comparison of pulmonary symptoms and function acute lymphoblastic leukaemia (ALL) survivor risk subgroups and controls with at least one valid pulmonary function test (PFT)

	Controls	ALL survivors				p-value
		SR	IR	HR	HR-SCT	
**≥1 valid PFT, age <18 years**	209	78	71	17	19	
**≥1 valid PFT, age 15–17.9 years**	34	6	9	7	5	
**Proxy-reported symptoms, age <18 years** ^#^	208	78	71	17	19	
Resting dyspnoea	0 (0.0)	1 (1.3)	0 (0.0)	1 (5.9)	1 (5.3)	0.010^##^
Exertional dyspnoea	7 (3.4)	8 (10.3)	10 (14.1)	3 (17.6)	5 (26.3)	<0.001^##^
**Self-reported symptoms, age 15–17.9 years** ^¶^	34	6	9	6	5	
Resting dyspnoea^+^	0 (0)	0 (0)	0 (0)	1 (17)	0 (0)	0.28^##^
Exertional dyspnoea^+^	0 (0)	0 (0)	0 (0)	2 (33)	0 (0)	0.023^##^
**Proxy-reported symptoms** ^#^	7	8	10	3	5	
Exertional dyspnoea, degree 1–5^§^	1.9±0.7	2.6±0.5	3.1±0.9	3.3±0.6	3.0±0.7	0.010^¶¶^
**N_2_MBW, valid**	146	51	48	13	14	
LCI 2.5%^ƒ^	6.56±0.62	6.64±0.64	6.60±0.75	6.61±0.59	7.44±1.57	<0.001^¶¶^
LCI 2.5% z-score^ƒ^	0.52±1.75	0.57±1.95	0.62±2.02	0.71±1.56	3.31±5.38	<0.001^¶¶^
**Spirometry, valid**	205	76	68	13	18	
FEV_1_ z-score	0.28±1.06	0.09±1.02	0.23±1.16	−0.37±0.85	−1.64±0.92	<0.001^¶¶^
FVC z-score	0.20±0.98	0.01±0.93	0.05±1.17	−0.67±0.93	−1.66±0.93	<0.001^¶¶^
FEV_1_/FVC z-score	0.12±1.02	0.11±1.02	0.30±0.99	0.55±0.72	0.02±1.42	0.42^¶¶^
**Spirometry with BDR**	192	65	56	10	16	
FEV_1_ % BDR	4.26±4.30	4.13±5.03	4.70±3.92	5.03±3.26	3.36±3.62	0.81^¶¶^
MMEF_75/25_ % BDR	13.60±12.41	15.83±18.75	13.29±12.26	18.40±9.85	15.25±17.19	0.65^¶¶^
**IOS, valid**	90	20	28	4	9	
*R*_5Hz_ z-score	−0.00±0.43	0.10±0.47	−0.00±0.50	−0.05±0.33	0.25±0.62	0.56^¶¶^
*X*_5Hz_ z-score	0.24±0.81	0.52±0.78	0.12±1.06	−0.22±0.81	−0.40±0.88	0.085^¶¶^
**IOS with BDR, valid**	68	15	25	2	4	
*R*_5Hz_ % BDR	−15.22±13.93	−18.67±8.14	−17.44±12.18	−27.50±7.78	−7.00±3.16	0.31^¶¶^
*X*_5Hz_ % BDR	−12.78±24.67	−20.60±14.36	−21.40±13.46	−25.00±16.97	−4.50±18.08	0.26^¶¶^
***D*_LCO_, valid**	119	39	30	8	10	
*D*_LCO_ z-score	0.12±0.90	−0.12±0.79	−0.22±0.89	−0.30±0.34	−1.23±0.68	<0.001^¶¶^
***D*_LNO_, valid**	99	37	25	7	9	
*D*_LNO_ z-score	0.97±2.79	−1.20±2.91	−1.53±2.48	−2.55±5.25	−2.39±1.26	<0.001^¶¶^
***D*_LNO_/*D*_LCO_, valid**	99	36	25	7	8	
*D*_LNO_/*D*_LCO_ ratio z-score	3.92±4.72	1.36±4.78	0.07±2.31	0.27±3.90	0.44±2.49	<0.001^¶¶^
**IOS, valid/IOS BDR, valid**	90/67	20/15	28/24	4/2	9/4	
*D*_5–20%_	9.20 (2.86–17.76)	19.82 (10.23–28.00)	14.31 (7.00–21.33)	12.40 (9.32–16.21)	22.07 (2.37–34.25)	0.058^++^
*D*_5–20%_ % BDR	−17.00 (−68.00–34.00)	−21.00 (−50.00–0.00)	−20.50 (−44.50–51.50)	−53.50 (−100.00– −7.00)	32.50 (−1.50–94.00)	0.31^++^

Data are presented as n, n (%), mean±sd or median (interquartile range), unless otherwise stated. SR: standard risk; IR: intermediate risk; HR: high risk; HR-SCT high-risk chemotherapy followed by stem cell transplantation; N_2_MBW: nitrogen multiple-breath washout test; LCI: lung clearance index; FEV_1_: forced expiratory volume in 1 s; FVC: forced vital capacity; BDR: bronchodilator response; MMEF_75/25_: forced expiratory flow between 25% and 75% of FVC; IOS: impulse oscillometry; *R*_5Hz_: airway resistance at a frequency of 5 Hz; *X*_5Hz_: airway reactance at a frequency of 5 Hz; *D*_LCO_: diffusing capacity of the lung for carbon monoxide; *D*_LNO_: diffusing capacity of the lung for nitric oxide; *D*_5–20%_: the difference between *R*_5Hz_ and *R*_20Hz_. ^#^: responses provided by a parent/caregiver for participants aged <18 years; ^¶^: responses provided by participants aged 15–17.9 years; ^+^: percentage of participants aged >15 years with at least one valid lung test and questionnaire; ^§^: 1=not at all, 2=very little, 3=moderate, 4=severe, 5=extreme; ^ƒ^: pooled N_2_MBW data from Sentrysuite version 3.1.6, 3.2.1 and 3.3.1/3.3.2; ^##^: Fisher's exact test of equality; ^¶¶^: one-way ANOVA of equality across groups; ^++^: Kruskal–Wallis one-way ANOVA.

#### Proxy-report questionnaire (age <18 years)

Significant prevalence differences for resting dyspnoea were observed across all survivor risk subgroups and controls overall (p=0.010), with the highest prevalence among HR and HR-SCT survivors, although observed within small ALL survivor subgroups. Among the three ALL survivors with proxy-reported resting dyspnoea, the mean degree was moderate to severe.

**FIGURE 2:**
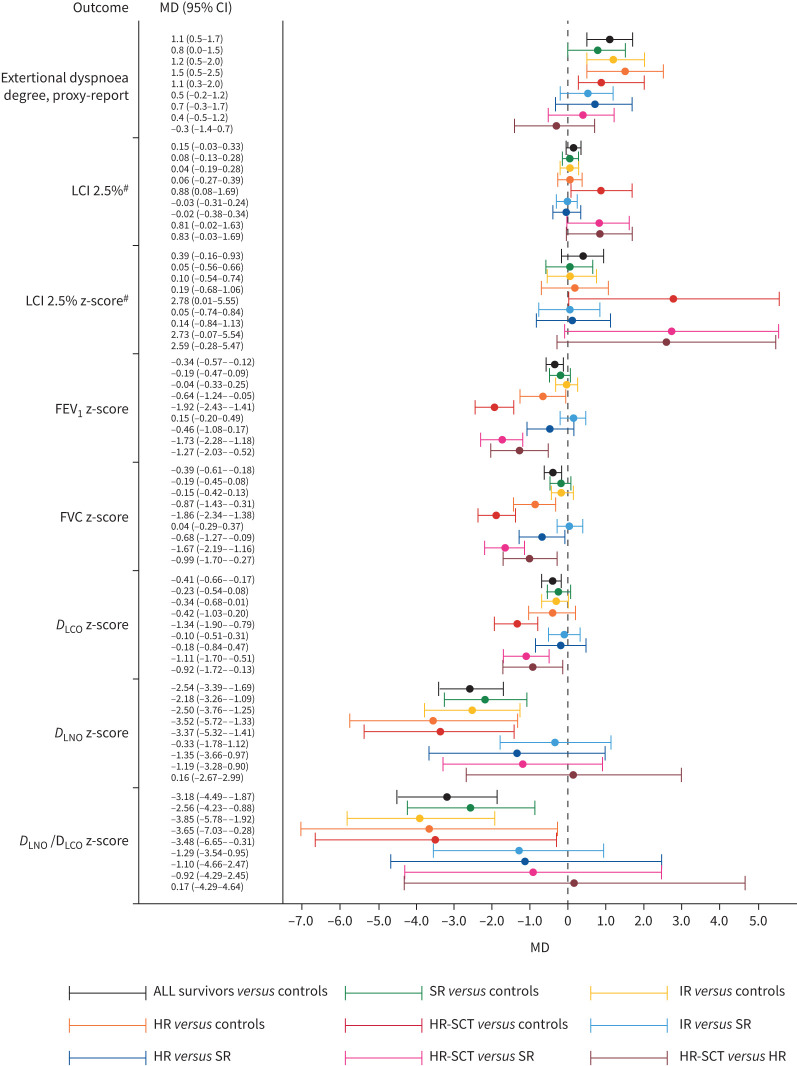
Comparison of pulmonary outcomes (exertional dyspnoea degree, parent proxy-reported and pulmonary function measures) across acute lymphoblastic leukaemia (ALL) survivors, ALL survivor final treatment risk subgroups (standard-risk (SR), intermediate-risk (IR), high-risk (HR), high-risk followed by stem-cell transplantation (HR-SCT)) and controls. Group comparisons are colour-coded, with mean differences (MD) and 95% confidence intervals provided for each parameter. For clarity, outcome measures are presented in sequential order from symptom (top) to specific pulmonary function parameters (bottom), as in table 3. Exertional dyspnoea degree: 1=not at all, 2=very little, 3=moderate, 4=severe, 5=extreme. LCI: lung clearance index; FEV_1_: forced expiratory volume in 1 s; FVC: forced vital capacity; *D*_LCO_: diffusing capacity of the lung for carbon monoxide; *D*_LNO_: diffusing capacity of the lung for nitric oxide. ^#^: pooled multiple breath washout data from Sentrysuite version 3.1.6, 3.2.1 and 3.3.1/3.3.2.

The prevalence of exertional dyspnoea in ALL survivors was significantly higher than among controls (p=<0.001), with the highest prevalences observed in HR and HR-SCT survivors. A significantly higher mean degree of exertional dyspnoea was observed in ALL survivors than in controls (p=0.001), with the highest mean degree in IR, HR and HR-SCT survivors (figure 2). Oxygen supply need was not reported in any group.

#### Self-report questionnaire (age 15–17.9 years)

Resting and exertional dyspnoea were rarely self-reported and only by HR survivors. Only HR survivors significantly differed from controls and the other risk groups (p=0.023). The degree of resting dyspnoea was reported as severe by one survivor, while exertional dyspnoea was rated moderate to severe.

### Pulmonary function

Among the participants, 185 ALL survivors and 192 controls had measurements from at least one valid PFT (table 2). We observed no difference in PFT measures regardless of dyspnoea status (resting and exertional), except for airway reactance at a frequency of 5 Hz (*X*_5Hz_). Survivors with exertional dyspnoea (proxy- and/or self-reported) demonstrated a significantly higher *X*_5Hz_ z-score (p=0.013) than asymptomatic survivors (table 4). A trend towards lower *D*_LNO_ z-score was observed in survivors with exertional dyspnoea than asymptomatic survivors (p=0.064).

**TABLE 4:** Comparison of pulmonary function among acute lymphoblastic leukaemia (ALL) survivors with at least one valid pulmonary function test (PFT) based on pulmonary symptom status

	Resting dyspnoea		p-value	Exertional dypsnoea		p-value
	No	Yes		No	Yes	
**ALL survivors,** ≥**1 valid PFT n=185**	182	3		159	26	
**N_2_MBW, valid**	123	3		110	16	
LCI 2.5%^#^	6.71±0.86	6.63±0.87	0.87^¶^	6.73±0.85	6.56±0.88	0.45^¶^
LCI 2.5% z-score^#^	0.92±2.67	0.59±2.30	0.83^¶^	0.98±2.69	0.41±2.37	0.42^¶^
**Spirometry, valid**	172	3		150	25	
FEV_1_ z-score	−0.06±1.19	−0.43±1.18	0.59^¶^	−0.05±1.18	−0.14±1.28	0.73^¶^
FVC z-score	0.21±1.04	0.29±0.98	0.89^¶^	0.26±1.05	−0.10±0.92	0.11^¶^
FEV_1_/FVC z-score	−0.19±1.15	−0.56±1.44	0.58^¶^	−0.20±1.17	−0.14±1.07	0.81
**Spirometry with BDR**	144	3		126	21	
FEV_1_ % BDR	4.32±4.41	4.30±2.04	0.99^¶^	4.37±4.52	4.06±3.39	0.77^¶^
MMEF_75/25_ % BDR	14.94±15.94	16.67±5.51	0.85^¶^	14.83±16.41	15.81±11.67	0.79^¶^
**IOS, valid**	59	2		52	9	
*R*_5Hz_ z-score	0.07±0.50	−0.03±0.59	0.80^¶^	0.04±0.51	0.22±0.41	0.30^¶^
*X*_5Hz_ z-score	0.17±0.97	−0.46±1.02	0.37^¶^	0.28±0.85	−0.57±1.29	0.013^¶^
**IOS with BDR, valid**	44	2		40	6	
*R*_5Hz_ % BDR	−17.32±10.58	−18.50±20.51	0.88^¶^	−17.67±10.71	−15.33±12.14	0.63^¶^
*X*_5Hz_ % BDR	−19.82±14.46	−20.00±24.04	0.99^¶^	−19.90±15.14	−19.33±11.22	0.93^¶^
***D*_LCO_, valid**	86	1		75	12	
*D*_LCO_ z-score	−0.28±0.84	−1.50	0.15^¶^	−0.24±0.83	−0.63±0.91	0.14^¶^
***D*_LNO_, valid**	77	1		66	12	
*D*_LNO_ z-score	−1.57±2.92	−1.23	0.91^¶^	−1.31±2.84	−2.99±2.94	0.064^¶^
***D*_LNO_/*D*_LCO_, valid**	75	1		64	12	
*D*_LNO_/*D*_LCO_ ratio z-score	0.66±3.78	6.45	0.13^¶^	0.86±4.05	0.11±2.18	0.53^¶^
**IOS, valid/IOS BDR, valid**	59/44	2/2		52/40	9/6	
*D*_5–20%_	16.11 (7.65–24.96)	3.72 (0.77–6.67)	0.082^+^	15.55 (7.61–24.83)	19.33 (6.84–24.51)	0.71^+^
*D*_5–20%_ % BDR	−20.00 (−43.00–19.00)	−27.00 (−100.00–46.00)	0.72^+^	−20.00 (−47.00–18.00)	5.50 (−36.00–46.00)	0.65^+^

Data are presented as n, mean±sd or median (interquartile range), unless otherwise stated. Resting dyspnoea and exertional dyspnoea cover both proxy- and self-reports combined (yes=proxy and/or self-reported dyspnoea, no=neither proxy- nor self-reported dyspnoea). N_2_MBW: nitrogen multiple-breath washout test; LCI: lung clearance index; FEV_1_: forced expiratory volume in 1 s; FVC: forced vital capacity; BDR: bronchodilator response; MMEF_75/25_: forced expiratory flow between 25% and 75% of FVC; IOS: impulse oscillometry; *R*_5Hz_: airway resistance at a frequency of 5 Hz; *X*_5Hz_: airway reactance at a frequency of 5 Hz; *D*_LCO_: diffusing capacity of the lung for carbon monoxide; *D*_LNO_: diffusing capacity of the lung for nitric oxide; *D*_5–20%_: the difference between *R*_5Hz_ and *R*_20Hz_. ^#^: pooled MBW data from SentrySuite version 3.1.6, 3.2.1 and 3.3.1/3.3.2; ^¶^: unpaired t-test of equality; ^+^: Wilcoxon rank sum test/Mann–Whitney U-test.

#### N_2_MBW

Only HR-SCT survivors demonstrated abnormal and significantly higher mean LCI 2.5% (p<0.001) and mean LCI 2.5% z-score (p<0.001) than controls. A trend for higher mean LCI 2.5% and LCI 2.5% z-score were observed for HR-SCT *versus* SR and HR, respectively (table 3, figure 2).

#### Spirometry with BDR

All survivor subgroups had mean forced expiratory volume in 1 s (FEV_1_) and forced vital capacity (FVC) z-scores within the standard normal range, except for HR-SCT survivors, whose FVC z-score and borderline FEV_1_ z-score were both very close to the lower limit of normal (LLN; z=−1.645) (table 3). However, FEV_1_ z-scores were significantly lower in ALL survivors than controls (p=0.003), particularly for HR and HR-SCT survivors. The FEV_1_ z-score in HR-SCT survivors was significantly lower than in SR and HR survivors (figure 2). The mean difference (95% CI) in FEV_1_ z-score was significant for HR *versus* controls. We observed significantly lower mean FVC z-score in survivors *versus* controls (p<0.001). The lowest FVC z-scores were observed in HR and HR-SCT survivors, the latter significantly lower than controls, SR survivors and even than HR survivors. For ALL survivors *versus* controls, FEV_1_/FVC z-scores (p=0.41), FEV_1_% BDR (p=0.88) and forced expiratory flow between 25% and 75% of FVC % BDR (p=0.37) were normal and comparable.

#### IOS with BDR

For airway resistance and reactance measures in ALL survivors *versus* controls, the mean airway resistance at a frequency of 5 Hz (*R*_5Hz_) z-scores (p=0.40) and *X*_5Hz_ z-scores (p=0.55) were within normal range and comparable (table 2). The median difference in airway resistance between 5 Hz and 20 Hz frequencies (*D*_5–20%_) was higher (but within normal range) in ALL survivors *versus* controls (p=0.009), with a trend towards significant differences across survivor risk subgroups and controls overall (p=0.058). IOS BDR revealed within normal range and comparable changes for survivors *versus* controls in mean *R*_5Hz_ (p=0.38), *X*_5Hz_ (p=0.084) and median *D*_5–20%_ (p=0.69). The only exception was an abnormal median (IQR) *D*_5–20%_ BDR for HR survivors (table 3).

#### *D*_LCO_, *D*_LNO_ and *D*_LNO_/*D*_LCO_

Both mean single-breath (SB) *D*_LCO_ z-score (p<0.001) and *D*_LNO_-SB z-score (p<0.001) were reduced in ALL survivors *versus* controls (table 2). Although within the standard normal range, the lowest mean *D*_LCO_-SB z-score measure was observed in HR-SCT survivors, who also demonstrated significant mean z-score differences compared to both controls and HR survivors.

*D*_LNO_-SB mean z-scores were moderately low (abnormal) in HR survivors and mildly low (abnormal) in HR-SCT survivors (table 3). Furthermore, *D*_LNO_-SB z-scores were significantly lower in SR, IR, HR and HR-SCT survivors than in controls (figure 2).

When comparing mean *D*_LNO_/*D*_LCO_ ratio z-scores, survivors showed a significant reduction compared to controls (p<0.001). The lowest measures, although within the standard normal range, were observed in IR, HR and HR-SCT survivors. Similarly, these groups’ absolute *D*_LNO_/*D*_LCO_ measures remained within the normal range (<5).

## Discussion

### Summary of results

This study confirms that ALL survivors, in particular those treated with HR and HR-SCT protocols, reported a higher prevalence of exertional dyspnoea than controls and exhibited impaired pulmonary function compared to controls. Resting dyspnoea was almost exclusively reported in HR and HR-SCT survivors. However, the prevalence of resting dyspnoea and self-reported exertional dyspnoea in these survivor subgroups should be interpreted cautiously due to small subgroups with relatively high frequencies. Proxy-reported exertional dyspnoea was significantly more severe among ALL survivors *versus* controls, particularly among IR, HR and HR-SCT survivors. Survivors exhibited impaired pulmonary function compared to controls in LCI 2.5% z-score (HR-SCT), FEV_1_ z-score (HR and HR-SCT), FVC z-score (HR and HR-SCT), *D*_LCO_ z-score (HR-SCT), *D*_LNO_ z-score (HR and HR-SCT), *D*_LNO_/ *D*_LCO_ z-score (HR and HR-SCT) and abnormal median *D*_5–20%_ BDR (HR). Importantly, SR and IR survivors mainly demonstrated PFT measures similar to controls, except *D*_LNO_ and *D*_LNO_/*D*_LCO_. This is reassuring, as these groups represent the majority of ALL survivors. We observed no difference in PFT measures between survivors regardless of dyspnoea status, except significantly higher *X*_5Hz_ z-scores in survivors with exertional dyspnoea (proxy- and/or self-reported).

### Potential mechanisms of pulmonary damage

The observed prevalence of proxy-reported exertional dyspnoea in ALL survivors overall (14.1%) was comparable to reports from another childhood ALL survivor study (15.4%) [33]. The increased prevalence observed in HR-SCT survivors (26.3%) coupled with overall normal BDR suggests that possible nonasthmatic impairments may be associated with higher chemotherapy exposure and/or SCT. Besides being associated with reduced airway reactance (*X*_5Hz_) and a trend for lower *D*_LNO_ z-score, exertional dyspnoea may stem from deconditioning and/or obesity, reflected by higher BMI z-scores observed in survivors.

A higher LCI 2.5% z-score in HR-SCT survivors indicates increased ventilation inhomogeneity and small airway dysfunction, likely reflecting small airway damage. This is associated with persisting pulmonary symptoms in post-(HR-)SCT studies [7, 8]. Similarly, the observed higher *X*_5Hz_ values in survivors with exertional dyspnoea suggest altered airway reactance (elasticity), also indicative of small airway involvement. The reduced lung function, as evidenced by a lower FEV_1_ z-score, has also been demonstrated in in childhood cancer survivor studies. Reduced FVC, as we observed, has been associated with chemotherapy-induced interstitial damage and/or restrictive changes in in childhood cancer survivors and post-SCT [34–36].

Significant FEV_1_ BDR was not observed in our study, suggesting that reversible airflow obstruction was not predominant in this cohort. However, the observed positive trend towards abnormal *D*_5–20%_ BDR may reflect potential ongoing subclinical airway hyperreactivity, which, if left unmanaged, may predispose to lung function decline. In HR-SCT survivors, such hyperreactivity is hypothesised to stem from immune-mediated processes, including airway alloreactivity, which has been linked to graft-*versus*-host disease (GvHD) following SCT [7, 8, 37].

Previous studies also reported on *D*_LCO_ z-scores, mainly after (HR-)SCT [16, 38–40], but did not assess *D*_LNO_ as we did. We observed reduced *D*_LCO_ and reduced and abnormal *D*_LNO_ z-scores, suggesting early signs of potential alveolar membrane damage, critical for pulmonary gas exchange and associated with long-term impaired cardiorespiratory fitness [41]. The larger reduction in *D*_LNO_ compared to *D*_LCO_ indicates that gas exchange impairment is linked to damage in the pulmonary interstitial tissue (composed of supporting structures such as capillaries and connective tissue), which impacts alveolar and endothelial function. Furthermore, pulmonary function deficit has been correlated to decreased physical function in adult in childhood cancer survivors [36], and its cumulative burden increases with age in adult ALL survivors [6].

This study is the first to assess *D*_LNO_ and *D*_LNO_/ *D*_LCO_ ratio in ALL survivors, providing new insights into gas exchange impairments. Furthermore, our study is the first to determine N_2_MBW, IOS and *D*_LNO_ in combination with symptomatology, spirometry and *D*_LCO_ in a large paediatric ALL survivor cohort. Notably, most ALL survivors (SR and IR) demonstrated pulmonary outcomes comparable to healthy controls, consistent with existing knowledge [35]. Our findings support the established risk of late pulmonary adverse effects from ALL treatment, affecting (small) airways, alveoli and potentially the interstitial tissues in a defined subgroup (HR and HR-SCT). Knowledge of pulmonary histopathological mechanisms in ALL survivors is limited due to the absence of studies involving lung biopsies. However, PFT assessment indirectly offers insights into interstitial changes and damage.

As demonstrated in our study, LCI 2.5% was the only PFT exclusively abnormal in HR-SCT survivors (moderately abnormal mean z-score), the sole group exposed to established pulmotoxic treatments (total body irradiation (TBI) and SCT), who also exhibited significantly higher prevalence of both resting and exertional dyspnoea and significantly impaired measures across all PFTs compared to controls. This highlights the crucial role of LCI screening in detecting small airway dysfunction and addresses the outstanding question in international recommendations regarding the impact and benefit of MBW in detecting early stages of pulmonary function deficit in children and young adult cancer survivors [17].

### Clinical relevance

Understanding the mechanisms of pulmonary damage is crucial for interpreting clinical relevance, as many cases may be treatable or preventable, and for enhancing follow-up recommendations. Although curative treatments for established pulmonary damage remain limited, early identification and management, such as the use of anti-inflammatory agents (corticosteroids and leukotriene receptor antagonists), bronchodilators or pulmonary rehabilitation, may alleviate symptoms, improve pulmonary outcomes and reduce the progression of damage [42]. While high ALL survival rates have been achieved through intensified regimens, the significant risk of long-term pulmonary complications underscores the need for mitigation strategies. Optimising chemotherapy protocols and supportive care, as well as balancing chemotherapy exposure with less toxic HR-SCT conditioning regimens can help reduce pulmonary toxicity. Early detection through baseline PFT and tailored monitoring are recommended. Certain chemotherapeutic drugs [43], TBI and SCT-associated GvHD can induce lung damage, manifesting as obstructive, restrictive and/or diffusion defects detectable by FEV_1_, FVC and *D*_LCO_/*D*_LNO_. *D*_LNO_ and the *D*_LNO_/*D*_LCO_ ratio provide added detail on the alveolar–capillary function and distinguishing between possible pulmonary and vascular sequelae [44].

The Children's Oncology Group accordingly recommends baseline spirometry and *D*_LCO_ for long-term follow-up [18], while the International Late Effects of Childhood Cancer Guideline Harmonization Group recommend a more conservative approach, limiting testing to symptomatic survivors at-risk (HR-SCT and TBI treated) due to limited evidence supporting testing asymptomatic survivors [17]. As we demonstrated, Nordic and Baltic paediatric and adult haematologists should remain vigilant about the late pulmonary adverse effects risk in NOPHO ALL2008 HR and HR-SCT survivors regardless of dyspnoea symptoms. Baseline PFTs at treatment cessation and follow-up are crucial. We recommend including baseline LCI because of its effort-independent advantage in younger children and its sensitivity to early detection of even subtle ventilation inhomogeneity, as well as *D*_LCO_ at the end of maintenance therapy. Similarly, LCI and *D*_LCO_ are recommended before and after HR-SCT treatment. Referral to a paediatric pulmonologist is advisable for survivors with pulmonary symptoms, abnormal PFT or pulmonary complications and should include patient education on symptom awareness [18, 45]. Moreover, clinicians should be aware that late pulmonary adverse effects, including exertional dyspnoea, may persist subclinically until a significant decline in pulmonary function becomes detectable, potentially leading to respiratory morbidity (*e.g.* lung fibrosis, bronchitis, emphysema) [46, 47] and delayed symptom onset.

### Strengths and limitations

The main strengths of this study include the use of a nationwide cohort of uniformly treated ALL survivors, the combination of subjective and objective pulmonary outcomes, a matched control population and a high recruitment rate (>70%), which minimises selection bias. Other strengths include height-, gender- and age-adjusted z-scores for PFTs, reducing confounding risk and enhancing the interpretability of the results, thereby mitigating minor demographic group differences regarding height and BMI. These strengths render our results applicable to all NOPHO ALL2008 survivors aged <18 years treated in Nordic and Baltic centres. Limitations include a relatively short and nonuniform follow-up period, which might not acknowledge the entire burden of ALL treatment. Furthermore, static lung volumes were not assessed, which could have provided further insight into potential restrictive lung impairments. Given that restrictive changes are generally expected to be more common than obstructive impairments in this patient group, future studies should include static lung volume assessment (*e.g.* body plethysmography) to provide a more comprehensive evaluation of pulmonary function in childhood ALL survivors. Another limitation was the known challenge of obtaining valid PFTs in younger patients, potentially leading to their under-representation in our analysis. Considering the potential vulnerabilities in younger *versus* older paediatric cancer survivors due to ongoing lung development, this under-representation may have led to an underestimation of pulmonary function impairments in ALL survivors and restricted the generalisability of our findings. Variations in software versions for LCI 2.5% and z-score reference equations may have introduced imprecision. Due to small sample sizes for HR and HR-SCT survivor subgroups, relatively high frequencies for proxy- and self-reported resting and self-reported exertional dyspnoea were observed within these groups. Therefore, these should be interpreted with caution. The lack of data on pre-existing lung disease (*e.g.* asthma) and medication use, which may have influenced pulmonary function and symptom reporting, represents a potential source of bias. Future non-cross-sectional studies with a causal aim should include these potential confounders for a more comprehensive understanding of factors contributing to late pulmonary adverse effects in childhood ALL survivors. Lastly, recruiting controls *via* ALL survivors and social media may have introduced self-selection bias, probably attracting more health-conscious participants than the general population. This could potentially have led to an overestimation of differences in pulmonary outcomes.

### Conclusions

In this nationwide study, HR and HR-SCT survivors reported a higher prevalence of exertional dyspnoea than controls, although observed within small subgroups. HR and HR-SCT survivors demonstrated impaired pulmonary function compared to controls, with indications of restrictive impairment, small airway dysfunction and alveolar damage, with less pronounced interstitial damage. However, the concern for late pulmonary adverse effects is primarily limited to these survivor subgroups and, importantly, most survivors do not exhibit significant late pulmonary adverse effects. Therefore, follow-up recommendations should include tailored, evidence-based strategies for clinicians to detect and monitor late pulmonary adverse effects early, beyond standard PFTs, focusing on HR and HR-SCT patients, regardless of symptoms, due to their increased vulnerability. Longitudinal studies are needed to identify predictors of late pulmonary adverse effects, allowing for more personalised follow-up recommendations and to improve long-term respiratory health outcomes in all childhood ALL survivors.

## Supplementary material

**Please note:** supplementary material is not edited by the Editorial Office, and is uploaded as it has been supplied by the author. Supplementary material 00350-2025.SUPPLEMENT
