# Supplementary material for: Late pulmonary adverse effects in childhood and adolescent acute lymphoblastic leukaemia survivors: a cross-sectional ALL-STAR Lungs study
Source: ERJ Open Res. 2025 Dec 1;11(6):00350-2025. doi: 10.1183/23120541.00350-2025 (PMC12683598; doi:10.1183/23120541.00350-2025)
Supplement: Supplementary file 1 [file 00350-2025.SUPPLEMENT.pdf]

Late Pulmonary Adverse Effects in Childhood and Adolescent Acute Lymphoblastic Leukaemia Survivors: A Cross-sectional ALL-STAR Lungs study

Sonja Izquierdo Riis Meyer, Mette Tiedemann Skipper, Birgitte Klug Albertsen, Ruta Tuckuviene, Peder Skov Wehner, Thomas Leth Frandsen, Kjeld Schmiegelow, Liv Andrés-Jensen, Kim Gjerum Nielsen, Sune Leisgaard Mørck Rubak

SUPPLEMENTARY MATERIAL

Methods

Variables and data sources

ALL treatment

Treatment regimens for the four ALL risk subgroups (standard risk (SR), intermediate risk (IR), high risk (HR) chemotherapy, and high risk chemotherapy followed by stem cell transplantation (HR-SCT)) under the NOPHO ALL2008 protocol were stratified based on the initial response to induction therapy - minimal residual disease (MRD) – and specific cytogenetic markers (Figure S1). SR and IR groups received less intensive chemotherapy without intensive high risk chemotherapy block treatments. In contrast, HR and HR-SCT groups underwent high-intensity regimens, including chemotherapy block treatments, with HR-SCT additionally subjected to conditioning regimens involving total body irradiation or myeloablative treatments [1]. These distinctions influence potential pulmonary toxicity risks, as HR and HR-SCT regimens involve higher cumulative doses of drug agents [2].

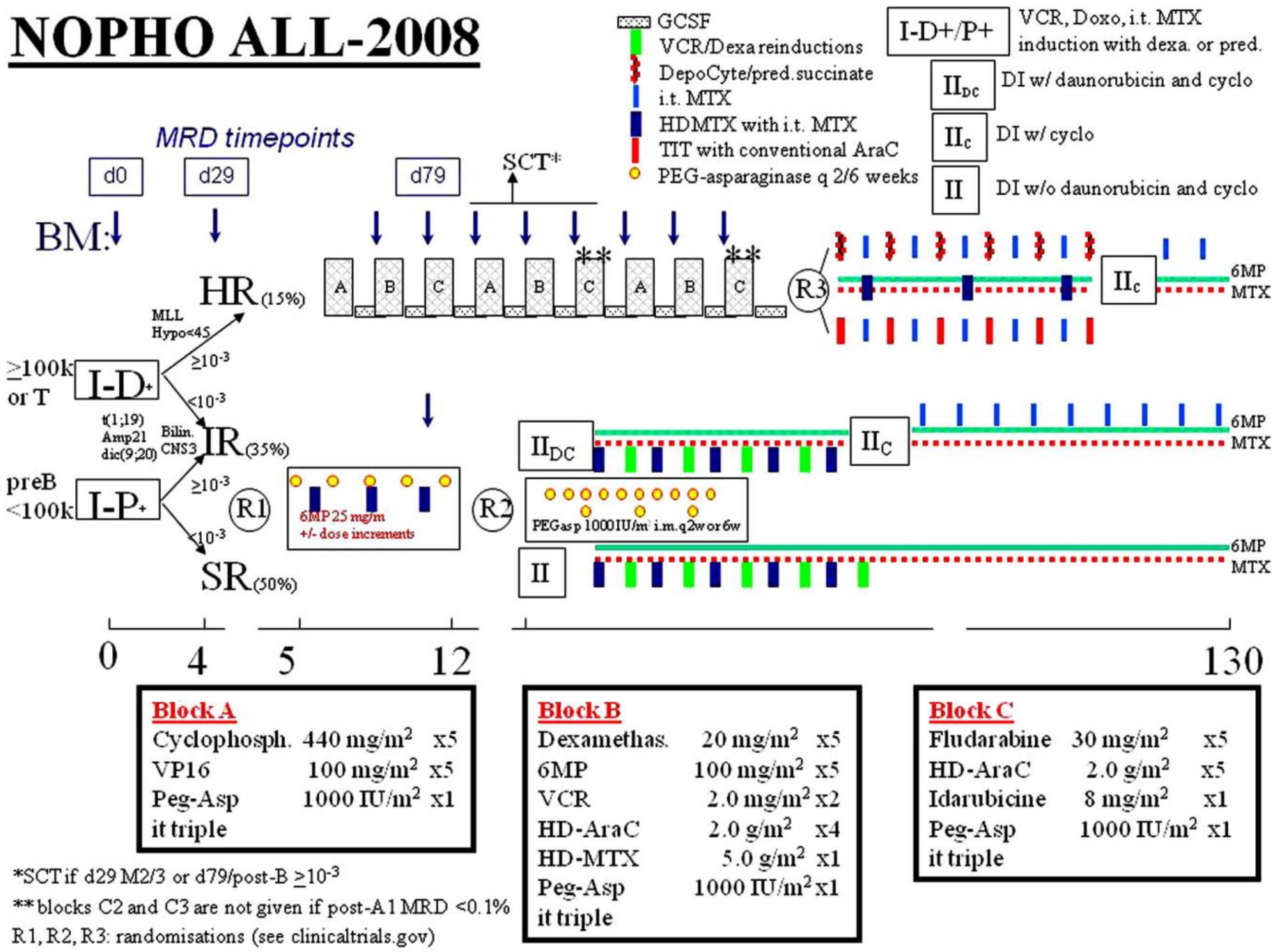

Figure S1

The NOPHO ALL-2008 protocol with stratifications and treatments. I-D+, induction therapy (Doxorubicin, Vincristine, i.t. Methotrexate) with Dexamethasone for High Risk Patients; I-P+, induction therapy (Doxorubicin, Vincristine, i.t. Methotrexate) with prednisolone for non-High Risk Patients. Stratification at day 29 to High Risk (HR), Intermediate Risk (IR) or Standard Risk (SR) according to response to treatment (Minimal Residual Disease, MRD) or specific cytogenetics. 3 randomisations are included in the protocol; R1, 6-mercaptopurine dose increments for non-HR patients (clinicaltrial.gov id number NCT00816049); R2, comparison of continuous versus intermittent PEG- asparaginase for non-HR patients (NCT00819351) and R3, standard triple intrathecal treatments versus intrathecal liposomal cytarabine with prednisolone for HR patients (NCT00991744) – R3 closed in October 2012 due to DepoCyte unavailability. Blocks A, B and C, high risk chemotherapy block treatments; IIDC, delayed intensification with Daunorubicin and Cyclophosphamide; IIC, Delayed Intensification with Cyclophosphamide; II, Delayed intensification without Daunorubicin and Cyclophosphamide; MTX, Methotrexate; HDM, High Dose Methotrexate; 6MP, 6-MercaptoPurine; VCR, Vincristine; SCT, stem cell transplantation; TIT, triple intrathecal Treatment with conventional cytarabine, prednisolone and methotrexate; GCSF, Granulocyte Colony Stimulating Factor.

Figure S1 and figure caption published by Frandsen TL et al., *Eur J cancer*, 2024 [1]. Reprint of figure and figure caption with permission through Rightslink/March 14, 2025.

Pulmonary outcomes

Blood samples were collected and analysed at Rigshospitalet or Aarhus University Hospital, Denmark (both laboratories accredited according to ISO 15189). Whole blood haemoglobin concentration was measured by Sysmex XN (XN, Sysmex, Kobe, Japan). See Table S1b for details of PFT variables, device, manufacturer and standard reference equations.

Nitrogen Multiple Breath Washout (N_2MBW) test

Nitrogen multiple breath washout test (N_2MBW) was performed to assess Lung Clearance Index (LCI) 2.5%, including quality control, following the ERS/ATS recommendations [3-6]. Tests were conducted using Exhalyzer D devices with Spiroware software. Real-time monitoring ensured appropriate tidal breathing patterns and a leak-free system during the measurement. Post-test data were reviewed for trial acceptability, requiring at least two reproducible and acceptable trials with functional residual capacity (FRC) variability within 25%. Retrospective validation included reanalysis of raw data with updated software to address calibration errors as described below. N_2MBW was assessed using different software versions at the two study sites (SPIROWARE 3.1.6, 3.2.1, 3.3.1 and 3.3.2). For older versions (3.1.6 and 3.2.1) raw N_2MBW data A-files were reloaded/reanalysed into the updated SPIROWARE version 3.3.1/3.3.2 with new dynamic delay synchronisation of flow, O_2 and CO_2 signals and calculation of the re-inspired N_2 volume (3.1.6) and correction of an earlier error in the cross-sensitivity correction for the O_2 and carbon dioxide gas sensors within the Exhalyzer D device (3.2.1, earlier resulting in an overestimation of N_2 concentration and N_2MBW outcomes, including LCI) [7]. N_2MBW raw data (A-files) were unavailable for a group of participants' N_2MBW tests. Therefore, we reported pooled SPIROWARE 3.1.6 and 3.3.1/3.3.2 LCI 2.5% and LCI 2.5% z-score data, as normalising LCI 2.5% data without available A-files was impossible.

Spirometry with Broncho Dilator Response (BDR)

Spirometry, including bronchodilator response (BDR), followed the 2019 ERS/ATS technical standards [8]. It included measurement of forced expiratory volume in the first second (FEV_1), including BDR, forced vital capacity (FVC), the ratio between FEV_1 and FVC (FEV_1/FVC) and forced expiratory flow between 25% and 75% of FVC BDR (MMEF75/25 BDR) [8]. Tests were repeated until three acceptable manoeuvres were obtained (maximum six trials) according to the standard criteria with variation in the two largest FVC values and the two largest FEV_1 values within 0.150 L or 5%. BDR testing followed bronchodilator administration and included repeated measures after a standardised delay [8]. Global Lung Function Initiative (GLI) 2012 standard

reference material was used [9], using Caucasian ethnicity, as this was most representative of the study population.

Impulse Oscillometry (IOS) with BDR

Impulse oscillometry (IOS) was performed only at Aarhus University Hospital. It adhered to the 2020 ERS guidelines [10] to assess airway resistance and reactance (elasticity) at a frequency of 5 Hz (R5Hz and X5Hz) during tidal breathing, the difference in airway resistance between 5 Hz and 20 Hz frequencies (D5-20%), including BDR for the same parameters (R5Hz BDR, X5Hz BDR, D5-20% BDR). At least three technically acceptable and reproducible trials (free of artefacts) were required with a coefficient of variability (CoV) <15% for children. BDR testing followed bronchodilator administration and included repeated measures after a standardised delay. Finally, we excluded data with coherence at 5 Hz < 0.8 at post-test evaluation.

Diffusing Capacity for Carbon Monoxide (DLCO)

Diffusing capacity for carbon monoxide (DLCO), corrected for haemoglobin, was assessed for real-time single-breath diffusion standardised by the 2017 ERS/ATS guidelines [11] to evaluate membrane diffusing capacity. Operators ensured appropriate inspiration depth and breath-hold time of 10±2 seconds. Post-test evaluation excluded data with inconsistent inspired volume (<90% of the largest vital capacity in the same test session), incomplete breath-hold time, evidence of leaks or Valsalvas/Müller manoeuvres during the breath-hold time or <85% of test gas volume inhaled in < 4 seconds. At least two acceptable DLCO measurements within 10% variability of each other and with a minimum of 4 minutes between tests were obtained. DLCO measures were collected in SI units (mmol/min/kPa) and converted into the traditional unit mL/min/mmHg by multiplying the values with 2.986421 before z-score calculations [12].

Diffusing Capacity for Nitric Oxide (DLNO)

Diffusing capacity for nitric oxide (DLNO) test followed the 2017 ERS/ATS standards [13]. Breath-hold times were set at 5–10 seconds, avoiding Valsalva or Müller manoeuvres to maintain stable intrathoracic pressures. Acceptable inspiration required at least 90% of inspiratory vital capacity in under 2.5 seconds. Post-test evaluation excluded tests with incomplete breath-hold times, leaks, or suboptimal inspiration. A minimum interval of 4 minutes between tests ensured complete clearance of prior test gases. At least two valid DLNO measurements with variability within 10% were required. DLNO measures were collected in SI unit (mmol/min/kPa).

**Table S1a.** Study objectives and outcome measures		
**Study objectives and outcome measures**		
**Questionnaire variables**	**Value**	**Details**
**Proxy-reported questionnaire (<18 years)**[i]		
Smoking status	Yes/No	Current smoking status
Resting dyspnoea	Yes/No	"Tendency to experience shortness of breath at rest?", "Is the symptom present now?"
Degree of resting dyspnoea	1-5	Impact degree[a] on everyday life: "How much does the shortness of breath affect your child's daily life?"
Exertional dyspnoea	Yes/No	"Does physical exertion cause shortness of breath in your child?", "Are exertion-related shortness of breath still present?"
Degree of exertional dyspnoea	1-5	Impact degree[a] on everyday life: "How much does the exertion-related shortness of breath affect your child's daily life?"
Need for oxygen supply	Yes/No	"Need for supplemental oxygen?", "Is the need for supplemental oxygen present now?"
Degree of oxygen need	1-5	Impact degree[a] on everyday life: "How much does the condition affect your child's daily life?"
**Self-reported questionnaire (15-17.9 years)**[j]		
Smoking status	Yes/No	Current smoking status
Resting dyspnoea	Yes/No	"Tendency to experience shortness of breath at rest?", "Is the symptom present now?"
Degree of resting dyspnoea	1-5	Impact degree[a] on everyday life: "How much does the shortness of breath affect your daily life?"
Exertional dyspnoea	Yes/No	"Does physical exertion cause shortness of breath in you?", "Are exertion-related shortness of breath still present?"
Degree of exertional dyspnoea	1-5	Impact degree[a] on everyday life: "How much do the exertion-related shortness of breath affect your daily life?"
Need for oxygen supply	Yes/No	"Need for supplemental oxygen?", "Is the need for supplemental oxygen present now?"
Degree of oxygen need	1-5	Impact degree[a] on everyday life: "How much does the condition affect your daily life?"
**Proxy-reported (<18 years)**[i]		
Smoking status	yes/no	Current smoking status
**Self-reported (15-17.9 years)**[j]		
Smoking status	yes/no	Current smoking status
**PFT variables**	**Unit**	**Details**
**N_2MBW**		Nitrogen multiple breath washout test
LCI 2.5%		Lung clearance index 2.5%
LCI 2.5% z-score		Lung Clearance Index 2.5% in z-score
**Spirometry with BDR**		Spirometry with bronchodilator response
FEV_1 z-score		Forced expiratory volume in the first second
FVC z-score		Forced vital capacity
FEV_1/FVC z-score		The ratio between FEV_1 and FVC
FEV_1 BDR[b]	%	BDR in forced expiratory volume in the first second

MMEF 75/25 BDR	%	BDR in forced expiratory flow between 25% and 75% of FVC
**IOS with BDR**		Impulse oscillometry with bronchodilator response
R5Hz z-score		Airway resistance at a frequency of 5 Hz
X5Hz z-score		Airway reactance at a frequency of 5 Hz
D5-20%	%	The difference between R5Hz and R20Hz
R5Hz % BDR[c]	%	BDR in airway resistance at a frequency of 5 Hz
X5Hz % BDR[d]	%	BDR in airway reactance at a frequency of 5 Hz
D5-20% % BDR[e]	%	BDR in the difference between R5Hz and R20Hz
**DLCO**		Diffusing capacity for carbon monoxide
DLCO z-score		Diffusing capacity for carbon monoxide in z-score
**DLNO**		Diffusing capacity for nitric oxide
DLNO z-score		Diffusing capacity for nitric oxide in z-score
**DLNO/DLCO**		DLNO/DLCO ratio
DLNO/DLCO z-score		The ratio between DLNO and DLCO in z-score
**Outcome measures**		
**Variables from clinical examination**	**Unit**	**Details**
**Anthropometrics**		
Height	m	Without shoes. For height z-score calculation[f]
Weight	kg	For BMI calculation
BMI	kg/m²	For BMI z-score calculation[g]
**Blood sample**		
Haemoglobin[h]	mmol/L	For haemoglobin corrected DLCO and DLNO

Abbreviations: m, meters; BMI, Body Mass Index; kg, kilograms; L, litres; BDR, broncho dilator response; FEV_1, forced expiratory volume in the first second; FVC, forced vital capacity; DLCO, diffusing capacity for carbon monoxide; DLNO, diffusing capacity for nitric oxide.

[a] degree: 1 = not at all, 2 = very little, 3 = moderate, 4 = severe, 5 = extreme.

[b] A ≥ 10% change (of the predicted value) in FEV_1 was considered a significantly abnormal BDR [48].

[c] A < -40% change in R5Hz was considered a significantly abnormal BDR [28].

[d] A > 50% change in X5Hz was considered a significantly abnormal BDR [28].

[e] A < -50% change in D5-20% was considered a significantly abnormal BDR. [f] National reference equations for height z-score [49]. [g] National reference equations for BMI z-score [50].

[h] Whole blood haemoglobin concentration was measured by Sysmex XN (XN, Sysmex, Kobe, Japan). Blood samples were collected and analysed at Rigshospitalet or Aarhus University Hospital, Denmark (both laboratories accredited according to ISO 15189).

[i] Proxy-reported: Responses provided by a parent/caregiver for participants aged <18 years.

[j] Self-reported: Responses provided by participants aged 15-17.9 years.

Table S1b. PFT devices and standard reference equations.

PFT and variables	Device	Manufacturer	Standard reference equations
**N_2MBW**			
LCI 2.5%, z-score	Exhalyzer D, N2 option	Eco Medics AG, Dürnten, Switzerland	Houltz et al., 2014 (preliminary)[a] [14]<br>Kentgens et al., 2022[b] [15]
**Spirometry**			
FEV_1, z-score	Jaeger MasterScreen Bodybox<br>Jaeger Vyntus Spiro	CareFusion, Hochberg<br>Vyaire Medical, Bayern	GLI: Quanjer et al., 2012[c] [9] and ERS/ATS: Stanojevic et al., 2022 [16]
FVC, z-score			
FEV_1/FVC z-score			
FEV_1 % BDR			
MMEF 75/25 BDR			
**IOS**			
R5Hz z-score	Jaeger MasterSceen Bodybox<br>Jaeger Vyntus IOS system	CareFusion, Hochberg<br>Vyaire Medical, Bayern	Nowowiejska et al., 2008 [17]
X5Hz z-score			
D5-20%			
R5Hz % BDR			ERS: King et al.., 2020 [10]
X5Hz % BDR			
D5-20% % BDR			
**DLCO and DLNO**			
DLCO-SB z-score	Jaeger Vyntus Body	CareFusion, Hochberg	ERS/ATS: Stanojevic et al., 2017 [12]
DLNO-SB z-score			Thomas et al., 2014 (GAMLSS) [18]
DLNO/DLCO z-score			

Abbreviations: PFT, pulmonary function test; N2MBW, nitrogen multiple breath washout test; LCI, Lung Clearance Index; FEV_1, forced expiratory volume in the first second; FVC, forced vital capacity; MMEF75/25, forced expiratory flow between 25% and 75% of FVC; BDR, broncho dilator response; GLI, The Global Lung Health Initiative; ERS, European Respiratory Society; ATS, American Thoragic Society; IOS, Impulse oscillometry; R5Hz, resistance at a frequency of 5 Hz; X5Hz, reactance at a frequency of 5 Hz; D5-20%, the difference between R5Hz and R20Hz; DLCO-SB, single breath diffusing capacity for carbon mono oxide; DLNO-SB, single breath diffusing capacity for nitric oxide; GAMLSS, Generalized Additive Models for Location Scale and Shape.

[a] For Spiroware version 3.1.6.

[b] For Spiroware version 3.3.1 and 3.3.2.

[c] Caucasian reference material.

Results

Other analysis

Subsequent analyses performed across subgroups of ALL survivors according to level of participation and validity of PFT and ALL-treated patients either deceased or lost to follow-up (LTFU) revealed statistically significant differences in mean ± SD age at examination and mean ± SD height (11.92 ± 2.95 vs 8.06 ± 2.12 vs 12.26 ± 3.19 years, p = .003 and 151.42 ± 17.20 vs 132.93 ± 11.72 vs 154.84 ± 17.09 cm, p = .014) overall. For mean ± SD time since diagnosis, the difference between groups was close to significant (7.15 ± 2.36 vs 5.20 ± 1.50 vs 7.77 ± 2.40 years, p = .057). Sub-analysis revealed that survivors with ≥ one valid PFT were older (11.92 ± 2.95 vs 8.06 ± 2.12 years, p = .001), taller (151.42 ± 17.20 vs 132.93 ± 11.72 cm, p = .005) and less recently diagnosed with ALL (7.15 ± 2.36 vs 5.20 ± 1.50 years, p = .032) than survivors with invalid PFTs (Table S2).

The groups were comparable overall regarding the distribution of final risk groups, gender, smoking status, mean height z-score, BMI, BMI z-score and age at ALL diagnosis. The results are provided in Table S2.

**Table S2.** Comparison of ALL survivors according to participation level and validity of PFT.							
	**ALL survivors**					**ALL treated**	
	**≧1 valid PFT**	**Invalid PFT(s)**	**No PFT done**	**Only survey and/or chart**	**Declined participation**	**Deceased or LTFU[f]**	
	**n**	**n**	**n**	**n**	**n**	**n**	
<18 years	185	7	14	41	48	23	
15-17.9 years	27	0	3	8	13	3	
	**n (%)**	**n (%)**	**n (%)**	**n (%)**	**n (%)**	**n (%)**	***p***
SR[f]	78 (42.2%)	5 (71.4%)	6 (42.9%)	20 (48.8%)	26 (54.2%)	6 (27.3%)	0.105[a]
IR[f]	71 (38.4%)	2 (28.6%)	7 (50.0%)	14 (34.1%)	15 (31.2%)	7 (31.8%)	
HR[f]	17 (9.2%)	0 (0.0%)	0 (0.0%)	1 (2.4%)	1 (2.1%)	7 (31.8%)	
HR-SCT[f]	19 (10.3%)	0 (0.0%)	1 (7.1%)	6 (14.6%)	6 (12.5%)	2 (9.1%)	
Male Sex	99 (53.5%)	4 (57.1%)	10 (71.4%)	17 (41.5%)	27 (56.2%)	8 (34.8%)	0.203[a]
Smoking, proxy-report[d]	3 (1.6%)	0 (0.0%)	1 (7.7%)	0 (0.0%)			0.360[a]
Smoking, self-report[e]	3 (12.0%)		0 (0.0%)	0 (0.0%)			1.000[a]
	**Mean (SD)**	**Mean (SD)**	**Mean (SD)**	**Mean (SD)**	**Mean (SD)**	**Mean (SD)**	***p***
Age at examination (years)	11.92 (2.95)	8.06 (2.12)	12.26 (3.19)				0.003[b]
Years since ALL diagnosis	7.15 (2.36)	5.20 (1.50)	7.77 (2.40)				0.057[b]
Height (cm)	151.42 (17.20)	132.93 (11.72)	154.84 (17.09)				0.014[b]
Height, z-score	-0.15 (1.14)	0.40 (1.32)	0.09 (0.84)				0.35[b]
BMI, z-score	0.41 (1.28)	1.27 (1.26)	0.83 (1.60)				0.13[b]
	**Median (IQR)**	**Median (IQR)**	**Median (IQR)**	**Median (IQR)**	**Median (IQR)**	**Median (IQR)**	***p***
Age at ALL diagnosis, years	4.08 (2.83, 6.12)	2.84 (1.88, 4.17)	2.97 (2.14, 7.28)	4.23 (2.79, 5.49)	4.01 (2.91, 6.43)	3.55 (2.16, 5.86)	0.31[c]
BMI (kg/m²)	18.00 (16.40, 20.50)	17.80 (16.10, 18.80)	19.20 (16.00, 22.70)				0.74[c]

Demographic data were missing regarding proxy-reported smoking status for 15 ALL survivors and self-reported smoking status (15-17.9 years) for nine ALL survivors participating with at least survey and/or chart.
Abbreviations: PFT, pulmonary function test; ALL, acute lymphoblastic leukaemia; SR, standard risk chemotherapy; IR, intermediate risk chemotherapy; HR, high risk chemotherapy; HR-SCT, high risk chemotherapy and haematopoietic stem cell transplantation; IQR, interquartile range; SD, standard deviation; n, number of study subjects; LTFU, lost to follow-up.
[a] Fisher's exact test of equality unadjusted for sex, age and height.
[b] One-way analysis of variance (ANOVA) of equality across groups unadjusted for sex, age and height.
[c] Kruskal-Wallis one-way analysis of variance unadjusted for sex, age and height.
[d] Percent of participants <18 years with proxy-reported smoking questionnaire: ≥1 valid PFT, n = 185; Invalid PFTs, n = 7; No PFT done, n = 13; Only survey and/or chart, n = 27.
[e] Percent of participants >15 years with self-reported smoking questionnaire: ≥1 valid PFT, n = 25; Invalid PFTs, n = 0; No PFT done, n = 2; Only survey and/or chart, n = 2.
[f] No risk group data were available for one deceased ALL patient.

References

1. Frandsen TL, Heyman M, Abrahamsson J*, et al.* Complying with the European Clinical Trials directive while surviving the administrative pressure - An alternative approach to toxicity registration in a cancer trial. *European Journal of Cancer* 2014; 50: 251-9.

2. Toft N, Birgens H, Abrahamsson J*, et al.* Toxicity profile and treatment delays in NOPHO ALL2008-comparing adults and children with Philadelphia chromosome-negative acute lymphoblastic leukemia. *Eur J Haematol* 2016; 96: 160-9.

3. Bhakta NR, McGowan A, Ramsey KA*, et al.* European Respiratory Society/American Thoracic Society technical statement: standardisation of the measurement of lung volumes, 2023 update. *Eur Respir J* 2023; 62.

4. Frauchiger BS, Carlens J, Herger A*, et al.* Multiple breath washout quality control in the clinical setting. *Pediatr Pulmonol* 2021; 56: 105-12.

5. Robinson PD, Latzin P, Ramsey KA*, et al.* Preschool Multiple-Breath Washout Testing. An Official American Thoracic Society Technical Statement. *American Journal of Respiratory and Critical Care Medicine* 2018; 197: e1-e19.

6. Robinson PD, Latzin P, Verbanck S*, et al.* Consensus statement for inert gas washout measurement using multiple- and single- breath tests. *European Respiratory Journal* 2013; 41: 507-22.

7. Wyler F, Oestreich MA, Frauchiger BS*, et al.* Correction of sensor crosstalk error in Exhalyzer D multiple-breath washout device significantly impacts outcomes in children with cystic fibrosis. *J Appl Physiol (1985)* 2021; 131: 1148-56.

8. Graham BL, Steenbruggen I, Miller MR*, et al.* Standardization of Spirometry 2019 Update. An Official American Thoracic Society and European Respiratory Society Technical Statement. *Am J Respir Crit Care Med* 2019; 200: e70-e88.

9. Quanjer PH, Stanojevic S, Cole TJ*, et al.* Multi-ethnic reference values for spirometry for the 3-95-yr age range: the global lung function 2012 equations. *Eur Respir J* 2012; 40: 1324-43.

10. King GG, Bates J, Berger KI*, et al.* Technical standards for respiratory oscillometry. *Eur Respir J* 2020; 55.

11. Graham BL, Brusasco V, Burgos F*, et al.* 2017 ERS/ATS standards for single-breath carbon monoxide uptake in the lung. *Eur Respir J* 2017; 49.

12. Stanojevic S, Graham BL, Cooper BG*, et al.* Official ERS technical standards: Global Lung Function Initiative reference values for the carbon monoxide transfer factor for Caucasians. *Eur Respir J* 2017; 50.

13. Zavorsky GS, Hsia CC, Hughes JM, *et al.* Standardisation and application of the single-breath determination of nitric oxide uptake in the lung. *Eur Respir J* 2017; 49.

14. Houltz BG, K.; Lindblad, A.; Singer, F.; Robinson, P.; Nielsen, K.; Gustafsson, P. Tidal N2 washout ventilation inhomogeneity indices in a reference population aged 7-70 years. 2012.

15. Kentgens AC, Latzin P, Anagnostopoulou P, *et al.* Normative multiple-breath washout data in school-aged children corrected for sensor error. *Eur Respir J* 2022; 60.

16. Stanojevic S, Kaminsky DA, Miller MR, *et al.* ERS/ATS technical standard on interpretive strategies for routine lung function tests. *Eur Respir J* 2022; 60.

17. Nowowiejska B, Tomalak W, Radliński J, *et al.* Transient reference values for impulse oscillometry for children aged 3-18 years. *Pediatr Pulmonol* 2008; 43: 1193-7.

18. Thomas A, Hanel B, Marott JL, *et al.* The single-breath diffusing capacity of CO and NO in healthy children of European descent. *PLoS One* 2014; 9: e113177.
